# Genetic Testing in Cystic Kidney Disease

**DOI:** 10.34067/KID.0000001127

**Published:** 2026-01-02

**Authors:** Jacqueline Soraru, Andrew J. Mallett, Hugh J. McCarthy, Amali Mallawaarachchi

**Affiliations:** 1 Department of Nephrology and Renal Transplantation, Fiona Stanley Hospital, Perth, Western Australia, Australia; 2 Department of Nephrology and Hypertension, Perth Children's Hospital, Perth, Western Australia, Australia; 3 Department of Nephrology, Townsville University Hospital, Townsville, Queensland, Australia; 4 College of Medicine and Dentistry, James Cook University, Townsville, Queensland, Australia; 5 Institute for Molecular Bioscience, The University of Queensland, Brisbane, Queensland, Australia; 6 Faculty of Medicine and Health, Sydney Medical School, University of Sydney, Sydney, New South Wales, Australia; 7 Centre for Kidney Research, Children's Hospital at Westmead, Westmead, New South Wales, Australia; 8 Department of Clinical Genetics, Royal Prince Alfred Hospital, Sydney, New South Wales, Australia; 9 Garvan Institute of Medical Research, Sydney, New South Wales, Australia

**Keywords:** ADPKD, genetic kidney disease

## Abstract

Genomic investigation is playing an increasing role in the management of cystic kidney diseases, reflecting a broader shift toward precision medicine in nephrology. Recent updates to the Kidney Disease Improving Global Outcomes Clinical Practice Guideline emphasize diagnostic genomics as a core component of autosomal dominant polycystic kidney disease care in particular, recognizing its utility across a range of clinical scenarios. Traditionally, diagnosis of autosomal dominant polycystic kidney disease has been clinical, using age-dependent imaging criteria for at-risk individuals *via* ultrasound and magnetic resonance imaging. Although these imaging modalities have good sensitivity, there are pitfalls in clinical diagnosis, particularly in patients with atypical clinical features, those without family history, or those at a young age. A confirmed genetic diagnosis can guide screening of at-risk family members, inform reproductive decisions, support safe selection of living related kidney donors, and provide the opportunity to use genotype-specific prognostication tools. In addition, as genotype-specific therapies enter the landscape, accurate genotyping will become essential for identifying which patients will benefit from treatment. This narrative review aims to provide a practical approach for the general nephrologist of when to offer genetic testing to patients with cystic kidney disease and outline the technical and genetic counseling considerations in the provision of patient-centered genetic investigation.

## Introduction

**Figure 1:**
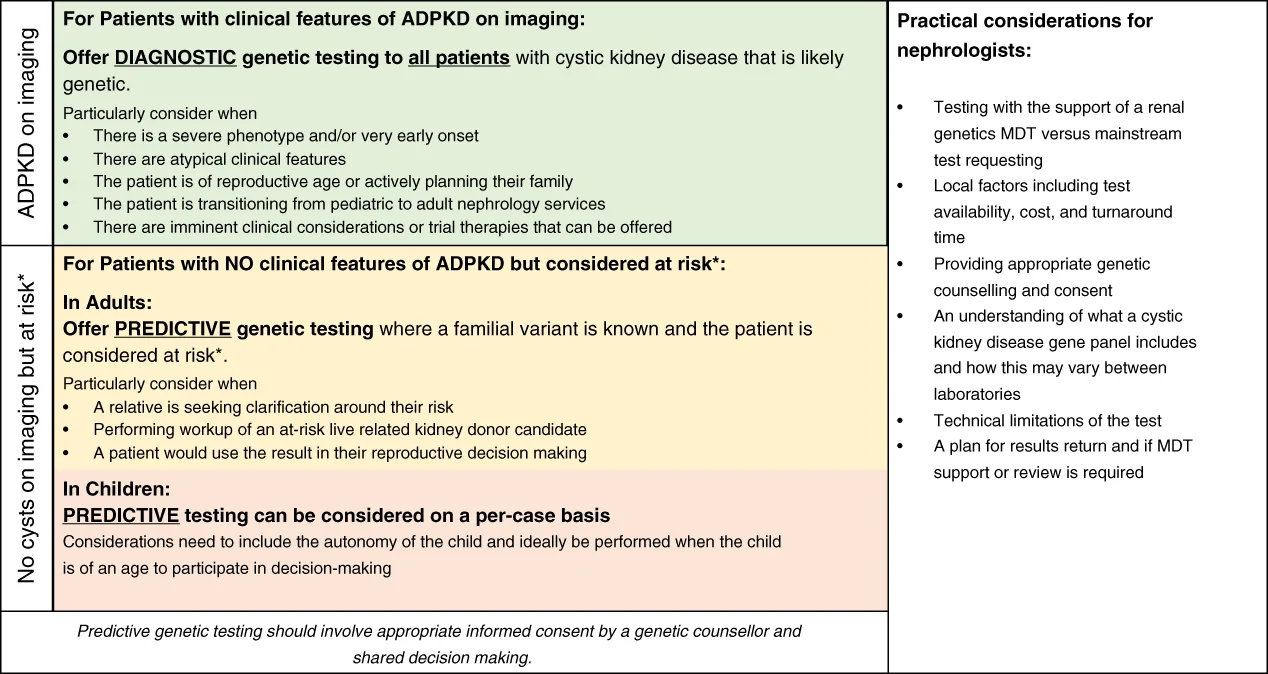
**Genetic testing in ADPKD.** An approach to genetic investigation in cystic kidney disease, including practical considerations for the nephrologist. *An individual is considered at risk of ADPKD if they have a first-degree relative with a clinical or molecular diagnosis of ADPKD. ADPKD, autosomal dominant polycystic kidney disease; MDT, multidisciplinary team.

Diagnosis of autosomal dominant polycystic kidney disease (ADPKD) has historically relied on clinical criteria, particularly imaging findings and family history.^1^ Although this approach has long served well, it has limitations. Recognizing this, the Kidney Disease Improving Global Outcomes (KDIGO) Clinical Practice Guideline highlights the role of genomics in the diagnostic workup of cystic kidney disease while recognizing the need for a structured approach.^2^ This shift is timely, because the genetic landscape of cystic kidney disease is becoming more complex at the same time as testing is becoming more readily available in mainstream practice. There are several aspects of cystic kidney disease genetics that make decisions around testing and result interpretation challenging. These include variable penetrance leading to different disease severity across generations, overlapping phenotypes with other cystic disorders, the presence of pseudogenes complicating analysis, challenges in variant interpretation, and the role of hypomorphic and mosaic variants on genotype-phenotype correlations. In addition, with the rapid evolution of sequencing technologies such as whole exome sequencing (WES), whole genome sequencing (WGS), and long-read sequencing, there are additional test sensitivity, specificity, and accessibility considerations. Establishing a structured, evidence-based framework for genetic testing in cystic kidney disease will enable consistent clinical practice and improve interpretation of genomic results (Figure 1).

### Genetic Diagnosis To Better Characterize Disease

ADPKD is defined as an autosomal dominant inherited condition primarily associated with kidney cysts.^2^ ADPKD exhibits a broad spectrum of clinical presentations, which can be differentiated on imaging features into typical or atypical disease. Typical ADPKD involves bilateral, diffuse, generally symmetrical cystic involvement of the kidneys with progressive nephromegaly.^2,3^ Atypical disease encompasses a heterogenous group of phenotypes that deviate from the classical ADPKD pattern. This includes focal or asymmetrical cystic disease, variable onset, without nephromegaly, and in some cases, with renal atrophy.^2^

**Figure 2:**
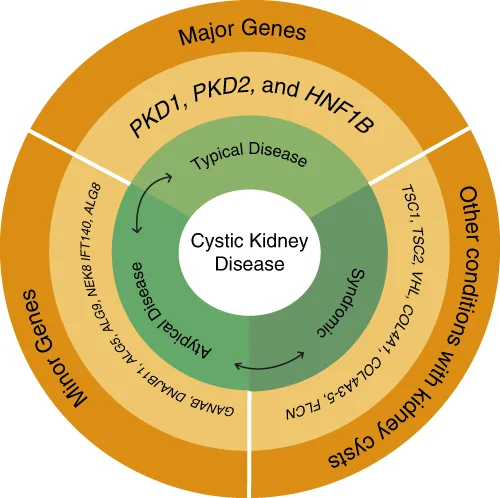
**Overview of cystic kidney disease.** Genes associated with typical, atypical, and syndromic cystic disease presentations, noting that this is not an exhaustive list. The green circle indicates the phenotype categories, the light orange middle circle indicates some of the genes associated with these phenotypes, and the outer dark orange circle indicates the gene categories.

There is value in genetic investigation in patients with both typical and atypical clinical presentations because the increasing number of ADPKD-associated genes makes it increasingly challenging to accurately distinguish the molecular cause on the basis of clinical and imaging features alone.^4^ In the past 10 years, there have been several genes recognized as associated with an expanded ADPKD phenotype. Disease-causing variants in *PKD1* and *PKD2* continue to account for the majority of typical ADPKD cases. However, disease-causing variants in cystic disease–associated genes such as *DNAJB11*, *GANAB*, *IFT140, ALG5, NEK8*, and *HNF1β* can produce ADPKD-like phenotypes.^4,5^ Kidney cysts can also occur in syndromic conditions, such as tuberous sclerosis complex (*TSC1* and *TSC2*),^6^ and tumor predisposition syndromes such as Birt–Hogg–Dube syndrome (*FLCN*) and Von Hippel-Lindau syndrome, and Alport syndrome (*COL4A3, COL4A4*, and *COL4A5*; Figure 2).^7^

Genetic investigation can better characterize the patient with cystic kidney disease, particularly those with atypical features and/or no family history, in which the cause of kidney cysts, and therefore treatment options and prognosis, are uncertain. Genetically differentiating the type of cystic kidney disease has implications for prognosis and screening for extrarenal manifestations. For example, patients with cystic kidney disease due to monoallelic *IFT140* loss of function variants, now thought to be the third most common cause of ADPKD, typically have conserved kidney function and are less likely to be hypertensive.^8–10^ Liver cysts are also rare.^8–10^ ADPKD-*GANAB* is associated with fewer cysts and a low rate of kidney failure but is commonly associated with polycystic liver disease.^11^ *DNAJB11* nephropathy typically involves nonenlarged or atrophic cystic kidneys, with interstitial fibrosis being the driving factor for kidney failure later in life.^12^ The phenotype is reminiscent of autosomal dominant tubulointerstitial kidney disease, and patients often have few to no liver cysts.^12,13^

Confirming a molecular diagnosis provides the opportunity to more specifically define ADPKD, with the recent KDIGO guideline suggesting that the genotype, where known, is used in the disease name (*e.g*., ADPKD-*PKD1* and ADPKD-*IFT140*).^2^ Correlation of genetic and clinical findings is key to defining disease. If a patient shows minimal signs (such as few kidney cysts) and lacks a family history of ADPKD, then identifying a genetic variant in an associated gene alone is not enough to confirm the diagnosis.^2^ This underscores the importance of interpreting genetic results as part of a patient's clinical picture, rather than in isolation.

### Prognostic Value of Genetic Testing

Genotype can assist in predicting outcomes. Differences in disease course and prognosis for ADPKD-*PKD1* and ADPKD-*PKD2* are well understood, with patients with *PKD2*-mediated disease less likely to have hypertension and urologic complications, and develop later-onset kidney failure.^14^ Clarity around prognosis in atypical ADPKD cohorts is increasing, and identifying a variant in a minor gene may reassure patients regarding their prognosis (Figure 2). This is particularly true for ADPKD-*IFT140* and ADPKD-*GANAB* that are associated with a milder renal phenotype.^9^ Other factors such as a patient's comorbid state and the trajectory of ADPKD within the family are important considerations in prognostication.

Differentiating a genetic cause helps identify patients in whom prognostication scores can be applied. Predicting renal outcome in PKD is a validated scoring tool that integrates genetic and clinical parameters including genotype (*PKD1* truncating versus nontruncating or *PKD2*), sex, age of onset of hypertension, and urologic events, to stratify patients' risk of progression to kidney failure.^15^ A higher score correlates with more aggressive disease, which can guide decisions around monitoring and therapeutics. The Mayo Imaging Classification system uses height-adjusted total kidney volume derived from computed tomography or magnetic resonance imaging to categorize patients into five subclasses (1A–1E), with a higher class conferring faster disease progression.^16^ The Mayo imaging criteria were validated in a population with mostly ADPKD-*PKD1* and ADPKD-*PKD2* diagnoses (92% confirmed, 3% no variant identified, and 5% not performed); therefore, there is value in understanding genotype before using these tools.^16^

Knowledge of the specific type of variant is also informative, with *PKD1* truncating variants conferring onset of kidney failure 12 years earlier than nontruncating *PKD1* variants^17^ and a 6× likelihood of kidney failure compared with a *PKD2* variant.^15^ Some nontruncating *PKD1* variants can still generate a significant amount of functioning protein and thus can be associated with mild ADPKD without risk of kidney failure.^18^

Given this genetic heterogeneity and genotype-dependent prognosis, all patients with typical or atypical cystic kidney disease suspected to be genetic should be offered genetic testing.

### Precision Therapy, Gene Therapy, and Clinical Trials

Genotype has implications for therapeutic management in ADPKD. Tolvaptan use is not predicated on a genetic diagnosis, although the original clinical trials were for patients with typical clinical features of ADPKD.^19^ The current KDIGO Guidelines recommend Tolvaptan in patients with ADPKD who are at risk of rapidly progressive disease on the basis of Mayo Imaging Classification or rate of eGFR decline.^2^ The predicting renal outcome in PKD score can be used as additional evidence for risk of progression in those with indeterminate eGFR or imaging criteria. Tolvaptan is not recommended for use in non-ADPKD–related cystic disease, such as due to *HNF1B*-disease or autosomal recessive polycystic kidney disease.^2^

Looking forward, gene therapies are encouraging in ADPKD, with clinical trials on the basis of promising mechanisms currently underway.^20–22^ These include a trial of an oligonucleotide inhibitor of a micro-RNA that targets *PKD1* (miR-17) and small molecule correctors of *PKD1* variants.^20,23^ Genetic diagnosis will likely be required for most of these gene-specific therapies, highlighting the importance of offering genetic testing to all patients with cystic kidney disease.^20^

ADPKD has been the focus of extensive clinical trial activity. Genotype-based stratification has the potential to enrich for individuals at higher risk of progression or better define disease subtypes, although it is currently not often incorporated within trial design. Considerations need to be made about the use of genotyping at the outset of a trial and whether this may limit access for those with less common genetic causes or reduce the generalizability of findings. An alternative approach is *post hoc* genetic stratification, which allows analysis of treatment response by genotype without restricting initial trial eligibility. This may help determine whether specific variants predict therapeutic response, while still ensuring broad participation. As gene-targeted and precision therapies continue to emerge, timely genetic diagnosis will support equitable access to research opportunities and future therapeutic innovation.

### Technical Considerations

Given the increasing number of ADPKD-associated genes being identified, the current mainstay of genetic investigation is based on gene panels. This is the concept of testing for all ADPKD-associated genes and possible differentials or phenocopies in a single genetic test. There are different sequencing options to consider, with the most commonly available testing options worldwide including targeted next generation sequencing (tNGS), WES, and WGS. When considering test selection, it is important to note the limitations of genetic testing, particularly in ADPKD. Six pseudogenes with remarkable sequence similarity to *PKD1* have been a longstanding technical challenge.^24^ The 2025 KDIGO Guideline recommends a testing approach that takes this into account. tNGS (with probes designed to avoid the pseudogenes) and WGS have robust evidence for coverage of the coding regions of the ADPKD genes.^25–27^ Of note, although used in large research analyses, standard WES that has not been enriched across *PKD1* has not been robustly validated as a diagnostic test for ADPKD and has technical limitations, meaning that this approach does not evenly cover the entire *PKD1* gene.^2,27^ Taking into account test cost and availability, the KDIGO recommendations are for initial investigation with a tNGS panel. If a patient remains negative with high clinical suspicion, WGS should be considered. WGS can identify splicing, noncoding, and regulatory region variants and is more robust for identifying deletions, duplications, and structural variants^28,29^ and, therefore, remains the single test most likely to identify all types of ADPKD variants.

There are also considerations for how a variant is classified. Some variants may be classified as a variant of uncertain significance and need further workup by the genomics laboratory or segregation among family members to clarify pathogenicity.

In addition to technical limitations, the yield of testing is reliant on appropriate test selection. Some gene panels are broader than others and will include phenocopy genes, which should be the preference if there are atypical features. There are varying levels of evidence for the association of a particular gene with a specific set of clinical features. Summaries of evidence for specific gene-disease associations are available through ClinGen, PanelApp-Australia, or Genomics England PanelApp, which publish publicly available curated panels of genes.^30,31^

When selecting a genetic test, clinicians should consider whether the breadth of the genes included covers the potential differentials, that the test has made considerations for *PKD1* pseudogene homology, can detect deletions and duplications, and is accredited as a clinical test. As with all genetic testing, it is important to understand that a negative or uncertain result does not exclude a genetic diagnosis. If there is a high clinical suspicion, referral to a research program or reanalysis as technology and knowledge advance is recommended. It is likely that emerging technologies, such as long-read sequencing, will increase diagnostic yield further.^28^

### Practical Approach to Genetic Testing

Increased knowledge and access to genetic testing for kidney disease has seen rapid growth in use by nephrologists. In an Australian study, 63% of Australian nephrologists or nephrology trainees had ordered a genomic test at least once.^32^ Referral to a renal genetics multidisciplinary team (MDT) was more common, with 95% of pediatric nephrologists and 75% of adult nephrologists referring for genetic investigation.^32^ Involvement of a renal genetics MDT can be pivotal in complex cases. Ideally, the MDT should involve a clinical geneticist, genetic counselor, diagnostic genomics laboratory staff, and a nephrologist, providing a multifaceted group to assess phenotype, carry out informed consent, identify appropriate testing pathways, and interpret results appropriately. For those without access to a renal genetics MDT, support can be sought from clinical genetics services.

Genetic testing can have broader implications compared with most other tests, particularly considerations for family members (including future and/or current children) and potential implications on insurability that vary between countries. These broad implications highlight the importance of genetic counseling as part of informed consent before genetic investigation and on returning genetic results. Informed consent involves a conversation with a willing and competent patient with time spent discussing the indication for testing, the type of test being requested, the possible outcomes of testing (including the possibility of uncertain or incidental findings), the plan for managing or delivering results, and the implication of results with regard to clinical management and at-risk family members. Before ordering genetic testing, the nephrologist should have an understanding of their local regulatory requirements regarding genetic investigation and consent and, if there are concerns, seek support from their local genetics service. In many regions, a signed genetic test consent form is required. Additional considerations include cost, with financial consent obtained if the test is ordered outside of a universal health care system.

### At-Risk Family Members

A positive genetic result in an affected individual may have implications for genetic relatives. Predictive testing, the practice of offering genetic testing for a disease-causing familial variant to asymptomatic at-risk relatives, may be considered for family members wanting to clarify their risk of ADPKD. The utility of this is context dependent—on the basis of factors such as the patient's drive for diagnosis, age, and reproductive stage. Given the specific considerations around predictive genetic testing, including insurance implications, predictive testing should be undertaken with informed consent *via* a genetic counselor. There are specific considerations for testing at-risk children (discussed later).

Where the disease-causing variant is known in a family, genetic testing of a related kidney donor candidate can exclude presymptomatic ADPKD. If the potential donor is <40 years with normal imaging, testing the potential donor for the familial variant can rule out the familial risk. If the donor is aged ≥40 years and there are no cysts on magnetic resonance imaging or computed tomography, imaging alone can exclude ADPKD, although genetically excluding a familial variant can increase the confidence of the donor in proceeding.^1^

### At-Risk or Affected Children

Genetic testing in children with cystic kidney disease can provide valuable diagnostic and prognostic information, particularly in differentiating between cystic dysplasia and primary cystic kidney disorders. Clarifying diagnosis may affect management and prognostication, alert to extrarenal manifestations, and clarify risk to relatives and potential living related donors. Offering testing in this situation involves discussion of the potential benefits of testing and should use a family-centered approach with age-appropriate discussions including the patient if they are able to participate.

In infants or young children with nephromegaly and overt cyst formation, genetic analysis can distinguish autosomal recessive polycystic kidney disease from biallelic or early-onset ADPKD, particularly where there is no family history.^33–35^ This distinction has implications for recurrence risk assessment and anticipatory care. If ADPKD is diagnosed, it may retrospectively trigger a diagnosis in a previously unknowingly affected parent.^33–35^

In children with a known affected parent, identification of a single cyst on imaging may be sufficient to confirm a clinical diagnosis, and hence the additional role of genetic testing in such cases is less clear.^1,2,36,37^ In a child with mild disease (*i.e*., a few typical cysts), performing a genetic test before young adulthood may add little to clinical management. If there are no immediate implications, deferring discussion about genetic testing to when the child is of an age to participate should be strongly considered. This time point generally coincides with transition from pediatric to adult services, and confirming a genetic diagnosis to risk stratify on the basis of genotype is increasingly important for young adults. This has management implications, particularly because treatments and clinical trial therapies are more easily accessible to young adults. Offering testing at this stage is also anticipatory for reproductive planning as patients enter reproductive years.

Predictive testing in asymptomatic, at-risk children remains complex, and practice varies. It can be considered on a per-case basis in families who are fully informed, highly motivated, and supported throughout by a genetic counselor, acknowledging the potential psychological and ethical complexities involved.^2^ Consideration should be given to the autonomy of the child, and in most cases, testing of an asymptomatic at-risk child can be deferred until the child is at an age that they can be involved in the decision-making process. In the meantime, follow-up of children at risk routinely involves annual BP assessment and urinalysis, with ultrasound and referral to pediatric nephrology warranted if hypertension or proteinuria develops.^38,39^

### Reproductive Decision Making

A genetic diagnosis provides families with information for their reproductive decision making. Utilizing reproductive technology, embryos derived from *in vitro* fertilization can be tested for the familial cystic kidney disease gene variant in a process known as preimplantation genetic testing (PGT).^40^ This requires prior confirmation of a disease-causing variant in the affected individual who is planning their family. PGT identifies affected and unaffected embryos and provides the opportunity to implant unaffected embryos, should they be available. Patients with ADPKD value the opportunity to use genetic testing in their family planning process—up to 50% of patients with CKD secondary to ADPKD would consider PGT, with this rising to 63% in those with kidney failure.^41^ This highlights the importance of nephrologists discussing genetic investigation and reproductive options with all patients with ADPKD who are family planning. Offering testing early is important, because the PGT process is often lengthy and involves dedicated genetic counseling and approval processes. PGT is not offered by all services, and familiarity with appropriate local referral pathways is required.

### Offer Genetic Testing to All Patients with ADPKD

Beyond impact on immediate clinical management, reproductive planning, and family screening, genetic diagnosis in cystic kidney disease holds an additional intrinsic value—providing patients with diagnostic clarity and bringing closure to a diagnostic odyssey (Table 1). The value of this is reflected in the recent KDIGO Guideline.^2^ Although not all patients may choose to undergo genetic testing, all should be offered the opportunity, using a context-sensitive approach that reflects local resources, test availability, and clinician expertise, while ensuring appropriate support to guide informed decision making.

**Table 1:** Indications for ADPKD genetic testing

Indication
Atypical clinical features
Diagnostic dilemma
Screening at-risk family members
Living related donor selection
Reproductive decision making
Guide treatment
Prognostication
Clinical trial eligibility

An adaptable factor in accessibility is the education and comfort level of the nephrologist in genetic investigation, with data showing that this is a key element of patients accessing testing.^32^ There are forums for nephrologists to increase their genomics knowledge, which is crucial to improving access for ADPKD families.^42^ Whether testing is initiated by the treating nephrologist, coordinated within a renal genetics MDT, or referred to a clinical genetics service, the goal is to ensure equitable access and informed uptake. As genotype-driven prognostication and therapies advance, genomic testing is poised to become integral to the standard of care in ADPKD. Facilitating access to testing today prepares families and clinicians to engage with the precision therapies of tomorrow.

## Supplementary Material
