# Supplementary material for: Genetic Testing in Cystic Kidney Disease
Source: Kidney360. 2026 Jan 2;7(8):1909–14. doi: 10.34067/KID.0000001127 (PMC13567892; doi:10.34067/KID.0000001127)
Supplement: Supplementary file 1 [file kidney360-7-1909-s001.pdf]

ASN American Society of Nephrology | JASN Journal of the American Society of Nephrology | KIDNEY360 Accessing Our World From Every Angle | CJASN Clinical Journal of the American Society of Nephrology

ASN Journal Disclosure Form

As per ASN journal policy, I have disclosed any financial relationships or commitments I have held in the past 36 months as included below. I have listed my Current Employer below to indicate there is a relationship requiring disclosure. If no relationship exists, my Current Employer is not listed.

A. Mallawaarachchi reports the following:
Employer: Garvan Institute of Medical Research; Consultancy: PYC Therapeutics; and Other Interests or Relationships: Scientific Advisory Board PKD Australia (not paid).

I understand that the information above will be published within the journal article, if accepted, and that failure to comply and/or to accurately and completely report the potential financial conflicts of interest could lead to the following: 1) Prior to publication, article rejection, or 2) Post-publication, sanctions ranging from, but not limited to, issuing a correction, reporting the inaccurate information to the authors' institution, banning authors from submitting work to ASN journals for varying lengths of time, and/or retraction of the published work.

Name: Amali Mallawaarachchi
Manuscript ID: K360-2025-000830R1
Manuscript Title: Genetic testing in cystic kidney disease
Date of Completion: October 26, 2025
Disclosure Updated Date: October 26, 2025

ASN Journal Disclosure Form

As per ASN journal policy, I have disclosed any financial relationships or commitments I have held in the past 36 months as included below. I have listed my Current Employer below to indicate there is a relationship requiring disclosure. If no relationship exists, my Current Employer is not listed.

A. Mallett reports the following:
Employer: Townsville University Hospital (Townsville Hospital and Health Service) and James Cook University; Research Funding: MRFF, Queensland Health, Australian Government (DoH), NHMRC; Advisory or Leadership Role: Natera and GSK (both unpaid advisory board), ANZSN Council (unpaid); and Other Interests or Relationships: Travel Support: Otsuka.

I understand that the information above will be published within the journal article, if accepted, and that failure to comply and/or to accurately and completely report the potential financial conflicts of interest could lead to the following: 1) Prior to publication, article rejection, or 2) Post-publication, sanctions ranging from, but not limited to, issuing a correction, reporting the inaccurate information to the authors' institution, banning authors from submitting work to ASN journals for varying lengths of time, and/or retraction of the published work.

Name: Andrew John Mallett
Manuscript ID: K360-2025-000830R1
Manuscript Title: Genetic testing in cystic kidney disease
Date of Completion: October 26, 2025
Disclosure Updated Date: June 18, 2025

ASN
American Society of Nephrology
JASN
Journal of the American Society of Nephrology
KIDNEY360
Accessing Our World From Every Angle
CJASN
Clinical Journal of the American Society of Nephrology

ASN Journal Disclosure Form

As per ASN journal policy, I have disclosed any financial relationships or commitments I have held in the past 36 months as included below. I have listed my Current Employer below to indicate there is a relationship requiring disclosure. If no relationship exists, my Current Employer is not listed.

H. McCarthy reports the following:
Employer: Sydney Childrens Hospitals Network; The University of Sydney; and Honoraria: AMGEN; Medisan.

I understand that the information above will be published within the journal article, if accepted, and that failure to comply and/or to accurately and completely report the potential financial conflicts of interest could lead to the following: 1) Prior to publication, article rejection, or 2) Post-publication, sanctions ranging from, but not limited to, issuing a correction, reporting the inaccurate information to the authors' institution, banning authors from submitting work to ASN journals for varying lengths of time, and/or retraction of the published work.

Name: Hugh J. McCarthy
Manuscript ID: K360-2025-000830R1
Manuscript Title: Genetic testing in cystic kidney disease
Date of Completion: October 30, 2025
Disclosure Updated Date: October 30, 2025

ASN American Society of Nephrology · JASN Journal of the American Society of Nephrology · KIDNEY360 Accessing Our World From Every Angle · CJASN Clinical Journal of the American Society of Nephrology

ASN Journal Disclosure Form

As per ASN journal policy, I have disclosed any financial relationships or commitments I have held in the past 36 months as included below. I have listed my Current Employer below to indicate there is a relationship requiring disclosure. If no relationship exists, my Current Employer is not listed.

J. Soraru reports the following:
Employer: Perth Children's Hospital; Fiona Stanley Hospital ; Sports Med Subiaco;; and Honoraria: Alexion.

I understand that the information above will be published within the journal article, if accepted, and that failure to comply and/or to accurately and completely report the potential financial conflicts of interest could lead to the following: 1) Prior to publication, article rejection, or 2) Post-publication, sanctions ranging from, but not limited to, issuing a correction, reporting the inaccurate information to the authors' institution, banning authors from submitting work to ASN journals for varying lengths of time, and/or retraction of the published work.

Name: Jacqueline Soraru
Manuscript ID: K360-2025-000830R1
Manuscript Title: Genetic testing in cystic kidney disease
Date of Completion: October 29, 2025
Disclosure Updated Date: October 29, 2025
